# Inhibiting β-Catenin by β-Carboline-Type MDM2 Inhibitor for Pancreatic Cancer Therapy

**DOI:** 10.3389/fphar.2018.00005

**Published:** 2018-01-17

**Authors:** Jiang-Jiang Qin, Wei Wang, Xin Li, Hemantkumar Deokar, John K. Buolamwini, Ruiwen Zhang

**Affiliations:** ^1^Department of Pharmacological and Pharmaceutical Sciences, College of Pharmacy, University of Houston, Houston, TX, United States; ^2^Center for Drug Discovery, University of Houston, Houston, TX, United States; ^3^Department of Pharmaceutical Sciences, College of Pharmacy, Rosalind Franklin University of Medicine and Science, North Chicago, IL, United States

**Keywords:** β-carboline, β-catenin, MDM2, p53, pancreatic cancer, protein degradation

## Abstract

The β-catenin and MDM2 oncoproteins are overexpressed and constitutively activated in human pancreatic cancer and contribute to its initiation, progression, and metastasis. The Wnt/β-catenin signaling pathway strongly interacts with the MDM2-p53 signaling pathway, accelerating the tumorigenesis and its development. Therefore, therapies inhibiting both β-catenin and MDM2 are suggested to be ideal treatments for patients with advanced pancreatic cancer. We have recently identified a novel class of β-carboline compounds as the specific and potent MDM2 inhibitors, including a lead compound SP141. In the present study, we utilized SP141 as an exemplary β-carboline compound to characterize β-catenin as a molecular target of the β-carboline compounds and to demonstrate an important role of β-catenin in the anticancer activity of β-carboline. We found that the silencing of either β-catenin or MDM2 largely reduced the anticancer activity of SP141 while the double silencing of both genes almost completely blocked SP141’s activity. SP141 directly bound to β-catenin and inhibited its expression and activity in pancreatic cancer cells *in vitro* and *in vivo*. The inhibitory effects of SP141 on β-catenin were mediated by the ubiquitin–proteasome system in an MDM2-independent manner. In conclusion, these results suggest that SP141 exerts its anticancer activity by dually inhibiting β-catenin and MDM2. We envision that β-carboline derivatives can be developed as promising dual inhibitors of β-catenin and MDM2 for the treatment of advanced pancreatic cancer.

## Introduction

The Wnt/β-catenin signaling pathway plays multiple regulatory roles in development and tissue homeostasis (Duan and Bonewald, 2016; Fujimura, 2016). Deregulation of Wnt/β-catenin signaling is implicated in various human cancers, including pancreatic cancer, and contributes to the initiation, progression, and metastasis of these diseases (Pai et al., 2016; Xue et al., 2016; Schneider and Logan, 2017). β-Catenin is a multifunctional protein, which exists in a dynamic mode at different subcellular localizations in cancer cells, including cell membrane, cytoplasm, and nucleus (Kim et al., 2013). The membrane-bound β-catenin protein directly interacts with E-cadherin, stabilizing cell–cell adhesion (Hulsken et al., 1994; Orsulic et al., 1999). The protein level of cytoplasmic β-catenin is tightly controlled by a destruction complex, comprising adenomatous polyposis coli (APC), Axin, casein kinase 1 (CK1), and glycogen synthase kinase 3β (GSK3β) (Stamos and Weis, 2013). The sequential phosphorylation of β-catenin by CK1 at Ser45 and by GSK3β at Ser33, Ser37, and Thr41 enables the recognition of β-catenin by β-transducin repeat-containing protein (β-TrCP), consequently resulting in the ubiquitination and proteasomal degradation of β-catenin (Yost et al., 1996; Orford et al., 1997; Stamos and Weis, 2013). Once dephosphorylated and translocated into nucleus, β-catenin directly binds to the T cell factor (TCF) transcription factors as well as the transcriptional co-activators, such as CREB-binding protein (CBP) and B cell lymphoma 9 (BCL-9), transcriptionally activating a broad spectrum of downstream target genes involved in cell proliferation, migration, apoptosis, and fate determination (Mosimann et al., 2009; Kim et al., 2013). Therefore, pharmacological inhibition of β-catenin activity and expression has been demonstrated as a promising approach for treating patients with advanced cancer, including advanced pancreatic cancer.

The Wnt/β-catenin pathway strongly interacts with the murine double minute 2 (MDM2)-p53 pathway during the initiation and progression of human cancer. It has been discovered that β-catenin overexpression stabilizes the p53 protein and increases its transcriptional activity by protecting it from MDM2-mediated proteasomal degradation (Damalas et al., 1999; Oren et al., 2002). However, the activated p53, in turn, downregulates β-catenin through CK1- and GSK3β-mediated phosphorylation as well as the ubiquitin–proteasome system (Sadot et al., 2001; Oren et al., 2002; Levina et al., 2004). The β-catenin C-terminus also represses p53 activity by an indirect mechanism (Riascos-Bernal et al., 2016). The deregulation of the Wnt/β-catenin signaling pathway in human cancer has been attributed to the inactivation of APC tumor suppressor (Sansom et al., 2004). A recent study has indicated that the ribosomal protein-MDM2-p53 pathway is another crucial mediator of APC loss-induced tumorigenesis (Liu et al., 2017). Herein, dual inhibition of β-catenin and MDM2 may display a synergistic anticancer activity and could be a promising therapeutic strategy for advanced pancreatic cancer.

We have recently discovered a novel class of β-carboline derivatives as small molecule inhibitors of the MDM2 oncoprotein. The lead compound SP141 has shown potent anticancer activity in breast and pancreatic cancer cells *in vitro* and *in vivo* (Wang et al., 2014b,c). We have demonstrated that SP141 directly binds to the MDM2 protein and induces its auto-ubiquitination and degradation, leading to cancer cell growth arrest and apoptosis in both p53-dependent and -independent manners (Wang et al., 2014b). In the virtual screening for ascertaining other molecular targets of SP141, we have discovered that β-catenin is one of the top candidate proteins. We have further shown that SP141 decreases the protein level of β-catenin and suppresses the tumor metastasis *in vitro* and *in vivo* (Wang et al., 2014b). This study is the first report that the β-carboline-type compounds can inhibit β-catenin for cancer therapy. The recent studies have shown the similar results that β-carboline derivatives can induce the degradation of β-catenin (Li et al., 2015; Ohishi et al., 2015). However, the detailed molecular mechanisms for the inhibitory effects of β-carbolines on β-catenin, especially the binding mechanisms are not fully elucidated. In the present study, SP141 was utilized as an exemplary β-carboline compound to investigate the molecular mechanisms of β-carboline’s inhibitory effects on β-catenin and assess the role of β-catenin in β-carboline’s anticancer activity. Further, it was examined whether MDM2 inhibition by SP141 was also involved in its inhibitory effects on β-catenin. These results might provide new insights into the drug design for developing novel β-catenin inhibitors for cancer therapy.

## Materials and Methods

### Chemicals, Antibodies, Plasmids, and siRNA

SP141 and biotinylated SP141 (biotin-SP141) were synthesized and purified as described previously (Wang et al., 2014b,c). Antibodies were purchased from BD Transduction (anti-β-catenin, 14/Beta-Catenin), Calbiochem (anti-MDM2, Ab-2), Santa Cruz (anti-c-Myc, 0.N.222; anti-cyclin D1, DCS-6; anti-Lamin B, C20), Sigma (anti-ubiquitin, 6C1; anti-β-actin, AC-15; anti-α-Tubulin, B-5-1-2), Cell Signaling (anti-phospho-β-Catenin, Ser33/37/Thr41), GeneTex (anti-phospho-β-Catenin, Ser45), Thermo Fisher Scientific (anti-6x-His, HIS.H8; anti-biotin, BTN.4), and Bio-Rad (goat anti-mouse IgG, H+L; goat anti-rabbit IgG, H+L). Plasmids expressing His-tagged full-length (Plasmid #17198), N-terminal (1-137; Plasmid #17203), and C-terminal (666-781; Plasmid #17204) human β-catenin were generated in Dr. Randall Moon’s lab (University of Washington) and purchased from Addgene. The Armadillo repeat and C-terminal (138-781) human β-catenin construct was kindly provided by Dr. Wenqing Xu (University of Washington). The siRNAs targeting β-catenin and MDM2 and the control siRNA were obtained from Thermo Scientific. Plasmids and siRNAs were transfected into the cells using the methods described previously (Wang et al., 2014a; Voruganti et al., 2015).

### Cell Culture and Cell Viability Assay

Human pancreatic cancer cell lines Panc-1 and AsPC-1 were purchased from American Type Culture Collection and cultured in RPMI 1640 medium supplemented with 10% FBS and 1% penicillin/streptomycin. The MTT assay was used to evaluate the effects of SP141 on pancreatic cancer cell viability as described (Qin et al., 2015a, 2016b). Briefly, cells were seeded in 96-well plates (3 × 10^3^ cells/well) and transfected with β-catenin siRNA, MDM2 siRNA, or both as indicated, followed by exposure to SP141 at various concentrations for 72 h. The treated cells were then incubated with MTT solution for an additional 3 h. Finally, the formazan crystals were dissolved in DMSO, and the absorbance was measured at 570 nm.

### Molecular Modeling

The molecular modeling of SP141-β-catenin binding was performed with the SYBYL-X 2.0 program package (Tripos), and the results were analyzed using Pymol 1.7 software. The crystal structure of β-catenin (PDB entry: 2Z6H) was used to generate the SP141-β-catenin binding complex. The SYBYL/Sketch module was applied to construct the structure of SP141, which was further optimized using Powell’s method and then assigned to SYBYL-X 2.0 software using the Gasteiger–Hückel method (Joshi et al., 2016; Chen et al., 2017).

### Streptavidin–Agarose Pull-Down Assay

The streptavidin–agarose pull-down assay was performed as described previously (Wang et al., 2014b). In brief, biotin-SP141 and biotin (negative control) were preincubated with streptavidin agarose beads (Invitrogen) overnight at 4°C. The biotin-SP141-conjugated beads were washed three times with cold PBS and then incubated with recombinant full-length or truncated β-catenin proteins or cell lysates overnight at 4°C, in the presence or absence of non-biotinylated SP141 (a binding competitor). The bead-bound proteins were boiled in SDS loading buffer and subjected to Western blotting.

### Western Blotting and Immunoprecipitation

Cells and tumor tissues were lysed in NP-40 lysis buffer containing protease inhibitors (Sigma). The lysates were evaluated for their protein concentrations using the Bradford reagent (Bio-Rad) and subjected to Western blotting as described previously (Qin et al., 2016a). Immunoprecipitation was performed to examine the ubiquitinated β-catenin as reported previously (Qin et al., 2015b). All the original images of the Western blots can be found in the Supplementary Material (Supplementary Figures 1–11).

### Real-Time Quantitative PCR

The total RNA was extracted from the control and SP141-treated cells using the Trizol reagent (Invitrogen) and the cDNA was synthesized by SuperScript reverse transcription-PCR (Invitrogen) according to the manufacturer’s protocols. The primer sequences were as follows: *c-Myc* forward, 5′-GCTGCTTAGACGCTGGATT-3′; *c-Myc* reverse, 5′-TGCTGCTGCTGGTAGAAGTT-3′; *CCND1* (*cyclin D1*) forward, 5′-GCATCTACACCGACAACTCC-3′; *CCND1* (*cyclin D1*) reverse, 5′-CGGATGATCTGTTTGTTCTCC-3′; *GAPDH* forward, 5′-GGAGTCCACTGGCGTCTTCAC-3′; *GAPDH* reverse, 5′-GAGGCATTGCTGATGATCTTGAGG-3′.

### Development and Treatment of Orthotopic Models of Pancreatic Cancer

The Panc-1 and AsPC-1 human pancreatic cancer orthotopic models were developed by following the animal study protocols that have been approved by the Institutional Animal Use and Care Committee of the Texas Tech University Health Sciences Center. The orthotopic tumor-bearing mice were treated with SP141 (i.p., 40 mg/kg/day, 5 days/week) or vehicle controls and monitored for tumor growth, changes in body weight, and other physical appearance as described previously (Wang et al., 2014c).

### Immunofluorescence and Immunohistochemistry

The cells on coverslips in a 12-well plate (1 × 10^4^ cells/well) were treated with SP141 for 12 h, followed by immunofluorescence detection as described previously (Qin et al., 2015b). The tumor tissues from the mice bearing Panc-1 and AsPC-1 orthotopic tumors were fixed and stained for the protein expression of β-catenin, c-Myc, and cyclin D1 following the reported protocols (Wang et al., 2014c).

### Statistical Analysis

All quantitative data were obtained from three or more independent experiments and expressed as mean ± SEM. Statistical significances between vehicle and treatment group were determined by Student’s *t*-test. A value of *P* < 0.05 was considered statistically significant.

## Results

### Both β-Catenin and MDM2 Are Essential for SP141’s Anticancer Activity

**FIGURE 1:**
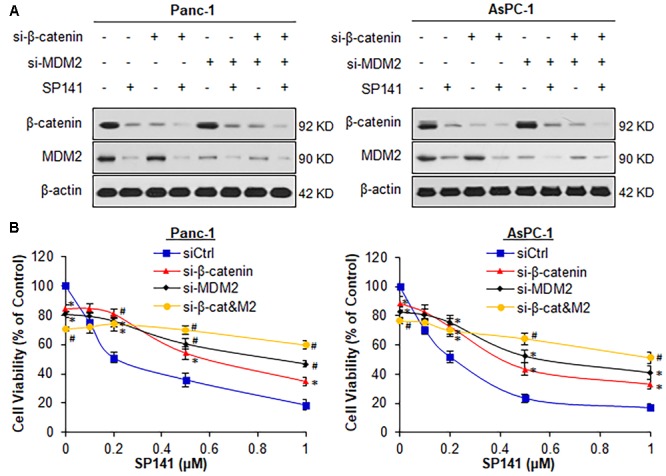
Both β-catenin and MDM2 are critical for SP141’s anti-pancreatic cancer activity. **(A,B)** Panc-1 and AsPC-1 cells were transfected with siRNAs targeting β-catenin and MDM2 or control siRNA, followed by treatment with SP141 at indicated concentrations for **(A)** 24 h for determination of the protein expression levels by Western blotting, and **(B)** 72 h for cell viability assay. All experiments were repeated at least three times, and similar results were obtained each time (^∗^*P* < 0.05, ^#^*P* < 0.01 vs. control siRNA groups).

To assess a role of β-catenin in SP141’s anticancer activity, we performed transient transfection experiments using β-catenin siRNA, MDM2 siRNA, or both. The pancreatic cancer Panc-1 (p53-mutant) and AsPC-1 (p53-*null*) cell lines were used in these studies because both cell lines harbor high expression levels of MDM2 and β-catenin, without wild-type p53. As examined by Western blotting and quantified using IMAGEJ software, the transfected cells exhibited a β-catenin and MDM2 knockdown (KD) efficiency of >60% (**Figure 1A**). MDM2 KD did not affect the endogenous β-catenin expression and did not alter the inhibitory effects of SP141 on β-catenin protein expression in both Panc-1 and AsPC-1 cells (**Figure 1A**). These results indicated that MDM2 was not involved in the inhibition of β-catenin by SP141. Similar results were obtained in β-catenin KD cells (**Figure 1A**).

We further examined the effects of the single KD of either β-catenin or MDM2 on SP141’s anticancer activity. As shown in **Figure 1B**, single KD of either β-catenin or MDM2 and double KD of both genes significantly reduced the viability of Panc-1 and AsPC-1 cells. β-Catenin KD markedly reduced SP141’s inhibitory effects on cell viability in comparison to cells transfected with control siRNA (**Figure 1B**). Consistent with our previous study (Wang et al., 2014c), MDM2 KD largely reduced SP141’s activity in pancreatic cancer cells (**Figure 1B**). The double KD of β-catenin and MDM2 almost completely blocked the inhibitory effects of SP141 on pancreatic cancer cell viability (**Figure 1B**).

### SP141 Decreases β-Catenin Expression and Activity in Pancreatic Cancer Cells *in Vitro* and *in Vivo*

**FIGURE 2:**
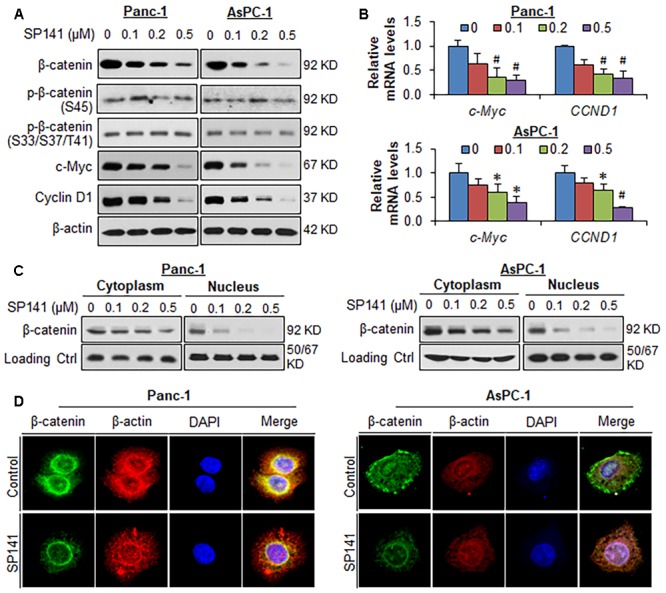
SP141 inhibits β-catenin expression and activity in pancreatic cancer cells *in vitro*. **(A)** Panc-1 and AsPC-1 cells were exposed to SP141 at indicated concentrations for 24 h. The expression of β-catenin, phosphorylated β-catenin at Ser45 and at Ser33/37/Thr41, c-Myc and cyclin D1 were examined by Western blotting. **(B)** Panc-1 and AsPC-1 cells were exposed to SP141 at indicated concentrations for 24 h. The relative mRNA levels of *c-Myc* and *CCND1* (*cyclin D1*) were determined by real-time quantitative PCR. **(C)** The cytoplasmic and nuclear fractions were extracted from SP141-treated Panc-1 and AsPC-1 cells using the NE-PER nuclear and cytoplasmic extraction kit. The distribution of β-catenin in the cytoplasm and nucleus was examined by Western blotting. α-Tubulin and Lamin B were used as loading controls, respectively. **(D)** Panc-1 and AsPC-1 cells were treated with SP141 for 24 h, followed by immunofluorescence detection. β-Actin and DAPI were used as internal references. The experiments were repeated at least three times, and similar results were obtained each time (^∗^*P* < 0.05, ^#^*P* < 0.01 vs. control).

We further examined the effects of SP141 on the expression, subcellular distribution, and activity of β-catenin in pancreatic cancer cells *in vitro*. As shown in **Figure 2A**, SP141 decreased the protein levels of β-catenin and its downstream target genes, c-Myc and cyclin D1 in both Panc-1 and AsPC-1 cells in a concentration-dependent manner. However, the compound did not change the expression levels of phosphorylated β-catenin proteins at Ser45 and at Ser33/37/Thr41. Similar observations were obtained with the decreased mRNA expression of both *c-Myc* and *CCND1* (*cyclin D1*) in Panc1 and AsPC1 cells in response to SP141 treatment (**Figure 2B**). It was further observed that SP141 largely reduced the β-catenin expression levels in nucleus (**Figure 2C**). An immunofluorescence assay was performed to detect the expression and distribution of β-catenin in SP141-treated cells. The results (**Figure 2D**) were similar to those observed by Western blot analysis. Of note, SP141 treatment did not affect the membrane-bound β-catenin protein.

**FIGURE 3:**
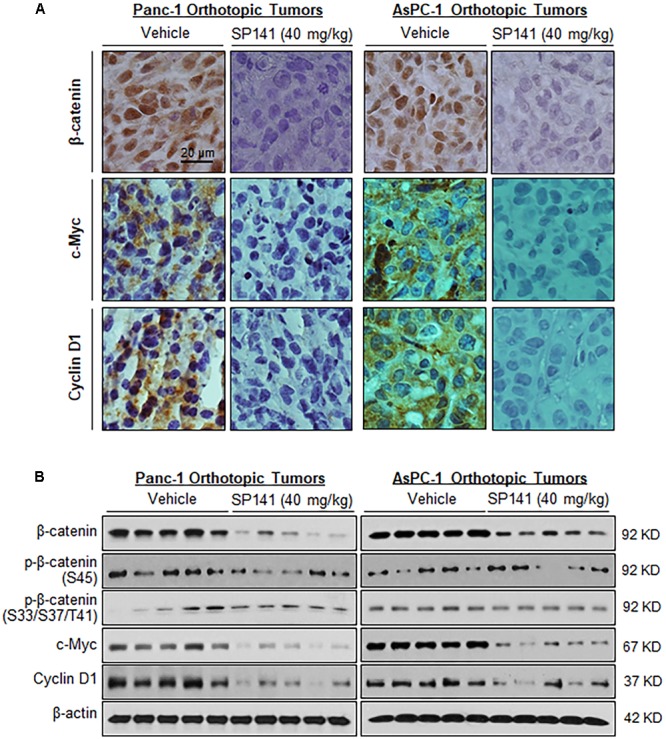
SP141 inhibits the expression of β-catenin and its target genes in pancreatic tumors *in vivo*. SP141 was administered by intraperitoneal injection (40 mg/kg/day, 5 days/week) to nude mice bearing Panc-1 and AsPC-1 orthotopic tumors for 5 and 4 weeks, respectively. The tumor tissues were analyzed for **(A)** the protein expression of β-catenin, c-Myc, and cyclin D1 by immunohistochemistry (scale bar: 20 μm) and **(B)** the protein expression of β-catenin, phosphorylated β-catenin at Ser45 and at Ser33/37/Thr41, c-Myc, and cyclin D1 by Western blotting (each lane represents a different tumor sample).

We also examined the effects of SP141 on the β-catenin expression in the tumors from pancreatic cancer orthotopic models that were used in the previous study (Wang et al., 2014c). In comparison to the vehicle-treated Panc-1 and AsPC-1 orthotopic tumors, a marked reduction of the expression of β-catenin and its targets c-Myc and cyclin D1 was observed in all the SP141-treated tumors, as determined by immunohistochemical staining (**Figure 3A**) and Western blotting (**Figure 3B**). Also, SP141 treatment did not affect the expression of phosphorylated β-catenin proteins at Ser45 and Ser33/37/Thr41 (**Figure 3B**).

### SP141 Directly Binds to β-Catenin Protein

**FIGURE 4:**
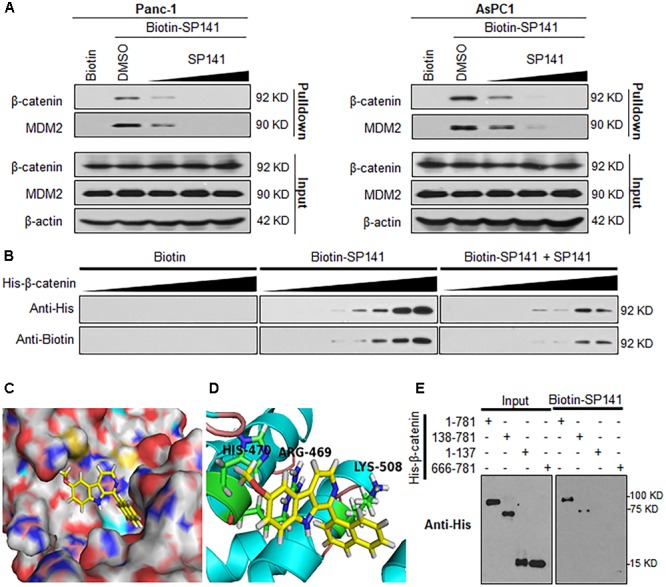
Identification of β-catenin as a target protein of SP141. **(A)** Biotinylated SP141 (biotin-SP141) was conjugated with avidin beads and then incubated with Panc-1 and AsPC-1 cell lysates in the presence or absence of 2-, 10-, and 20-fold excess of non-biotinylated SP141. The mixtures were blotted for bound β-catenin and MDM2 proteins. **(B)** Biotin-SP141-conjugated avidin beads were incubated with various concentrations of recombinant His-β-catenin in the presence or absence of non-biotinylated SP141. The bound proteins were detected using anti-His and anti-Biotin antibodies. **(C)** Computational modeling of SP141 binding to β-catenin. SP141 was rendered in yellow. **(D)** The predicted binding mode of SP141 with β-catenin. The key residues interacting with SP141 were rendered as sticks. **(E)** Biotin-SP141-conjugated avidin beads were incubated with the His-tagged recombinant proteins of full length and different regions of β-catenin. The inputs and bound proteins were examined using anti-His antibody. All experiments were repeated at least three times, and similar results were obtained each time.

To further understand the molecular mechanisms of SP141’s inhibitory effects on β-catenin, we performed a pull-down assay using biotinylated SP141 (biotin-SP141)-bound beads and pancreatic cancer cell lysates. As shown in **Figure 4A**, both the β-catenin and MDM2 proteins in the Panc-1 and AsPC-1 cell lysates were specifically precipitated by biotin-SP141, but not the biotin tag. We further observed that a preincubation of non-biotinylated SP141 significantly reduced the formation of SP141-β-catenin and SP141-MDM2 complexes in the cell lysates (**Figure 4A**). These results indicated that SP141 could directly bind to β-catenin protein, with an affinity similar to the SP141-MDM2 binding. Similar results were obtained with recombinant His-β-catenin protein. As shown in **Figure 4B**, an increase in the formation of SP141-β-catenin complex was observed with the increase of the preincubated His-β-catenin protein. The SP141-β-catenin complex was further reduced by a preincubation of the non-biotinylated SP141, suggesting a specificity of the SP141-β-catenin binding.

To further examine the binding mechanism of SP141 with β-catenin, we performed a molecular docking study. As shown in **Figures 4C,D**, SP141 could directly and specifically bind to the Armadillo repeat domain of β-catenin. The SP141-β-catenin binding mode indicated that the naphthyl group and methoxyl group of SP141 displayed hydrophobic interaction with LYS508 and HIS470 in the β-catenin, respectively (**Figure 4D**). The nitrogen at the 9-position of SP141 might directly interact with ARG469 via a hydrogen bond (**Figure 4D**). As shown in **Figure 4E**, SP141 directly bound to the full length β-catenin and the β-catenin construct harboring Armadillo repeat domain and C-terminal domain. However, the compound did not show any binding to the N-terminal and C-terminal domains of β-catenin. These results further validated a specific binding of SP141 to the Armadillo repeat domain.

### SP141 Induces β-Catenin Ubiquitination and Proteasomal Degradation

**FIGURE 5:**
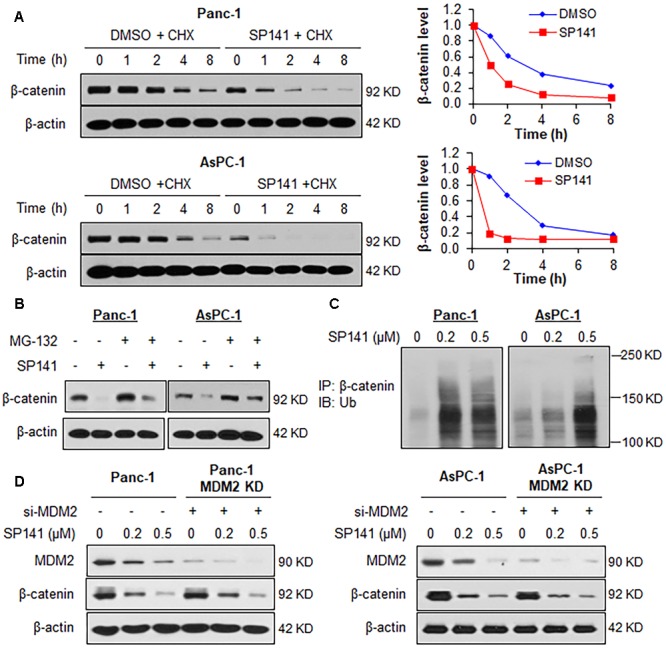
SP141 induces proteasomal degradation of β-catenin. **(A)** Panc-1 and AsPC-1 cells were exposed to SP141 (0.5 μM) for 24 h, followed by treatment with a protein synthesis inhibitor, cycloheximide (CHX, 15 μg/mL). The β-catenin protein expression at indicated times after exposure to CHX was determined by Western blotting. Graphs (on the right) show the quantification of immunoblotting data. **(B)** Panc-1 and AsPC-1 cells were treated with SP141 (0.5 μM) for 24 h, followed by exposure to a proteasome inhibitor MG-132 (25 μM) for 6 h. The β-catenin protein expression was determined by Western blotting. **(C)** Panc-1 and AsPC-1 cells transfected with ubiquitin plasmid were exposed to SP141 at indicated concentrations for 24 h, followed by an immunoprecipitation assay with an anti-β-catenin antibody. The ubiquitinated β-catenin was detected using an anti-ubiquitin antibody. **(D)** Panc-1 and AsPC-1 cells were transfected with siRNAs targeting MDM2 or control siRNA, followed by treatment with SP141 at indicated concentrations for 24 h for determination of the protein expression levels by Western blotting. All experiments were repeated at least three times, and similar results were obtained each time.

We further examined whether the binding of SP141 to β-catenin could induce its destabilization. As shown in **Figure 5A**, SP141 treatment shortened the half-life of β-catenin and accelerated its protein degradation in Panc-1 and AsPC-1 cells. SP141-induced destabilization of β-catenin was significantly inhibited by a proteasome inhibitor MG-132, suggesting that SP141 could enhance β-catenin degradation via the ubiquitin–proteasome pathway (**Figure 5B**). To test this hypothesis, we transfected the Panc-1 and AsPC-1 cells with an ubiquitin plasmid and treated the transfected cells with SP141. As shown in **Figure 5C**, a Western blot analysis of immunoprecipitated β-catenin exhibited a concentration-dependent enhancement of β-catenin ubiquitination by SP141 treatment. We further assessed a role of MDM2 in SP141’s inhibitory effects on β-catenin. As shown in **Figure 5D**, no significant change of SP141-induced β-catenin degradation was observed in both Panc-1 and AsPC-1 cells with or without MDM2 silencing. We, therefore, concluded that SP141 destabilized β-catenin via a proteasome-dependent and MDM2-independent pathway.

## Discussion

Cancer development and progression is a complex process that is driven by multiple molecular mutations and genetic abnormalities (Watson et al., 2013; Muller and Vousden, 2014; Hobbs et al., 2016). Currently, the majority of cancer therapeutics have been developed to target a single molecular target or pathway, such as β-catenin and MDM2 (Nag et al., 2013; Thakur and Mishra, 2013; Voronkov and Krauss, 2013; Lemos et al., 2016). However, the compensation for the target inhibition by other closely related pathways often causes inherent redundancy or acquired resistance, leading to the limited efficacy of these therapeutics (Holohan et al., 2013; Huang et al., 2014). The initiation, progression, and metastasis of human pancreatic cancer have been attributed to the mutation and deletion of multiple tumor suppressor genes (e.g., *APC, TP53*, etc.) (Duffy et al., 2014; Lesko et al., 2014) and the overexpression and aberrant activation of multiple oncogenes (e.g., *CTNNB1, MDM2*, etc.) (Qin et al., 2012; Thakur and Mishra, 2013; Voronkov and Krauss, 2013; Lemos et al., 2016). It has been demonstrated that the Wnt/β-catenin and the MDM2-p53 pathways have synergistic effects in cancer development and progression (Damalas et al., 1999; Oren et al., 2002; Liu et al., 2017). Therefore, dual targeting of the Wnt/β-catenin and the MDM2-p53 pathways may be a novel and promising approach for the treatment of pancreatic cancer.

The main aim of the present study was to investigate a role of β-catenin in the anticancer activity of a β-carboline-type MDM2 inhibitor SP141 as well as the molecular mechanisms that are responsible for the inhibition of β-catenin by SP141. In this study, we demonstrated that the inhibitory effects of SP141 on β-catenin played a crucial role in its anticancer activity, as evidenced by the β-catenin KD pancreatic cancer cell lines. Importantly, the double KD of β-catenin and MDM2 almost entirely blocked SP141’s anti-pancreatic cancer activity, indicating that the dual inhibition of β-catenin and MDM2 by this compound might exhibit a synergistic activity against tumor growth. It was further discovered that SP141 directly bound to the Armadillo repeat domain of β-catenin and inhibited its expression and activity in pancreatic cancer cells *in vitro* and *in vivo*. The binding of SP141 to β-catenin could cause its ubiquitination and proteasomal degradation. The SP141-induced β-catenin degradation occurred without affecting the expression level of phosphorylated β-catenin, which was distinct from previously reported small molecule inhibitors (Voronkov and Krauss, 2013). Furthermore, SP141-induced MDM2 degradation was not involved in β-catenin inhibition by SP141 because neither β-catenin KD nor MDM2 KD affected the expression of another gene in p53-mutant and p53-*null* pancreatic cancer cells.

To date, there is no clinically approved small molecule β-catenin inhibitor. The majority of previously reported β-catenin inhibitors have been developed to target the binding of β-catenin to TCF, CBP or BCL-9, inhibiting the transcription of its downstream target genes (Voronkov and Krauss, 2013). ICG-001 is a small molecule inhibitor that has been designed to inhibit the β-catenin-CBP interaction (Arensman et al., 2014). Its derivative PRI-724 is currently in phase Ib/IIa clinical trials (Bahrami et al., 2017). Many studies have also focused on targeting the destruction complex or other upstream regulators for β-catenin protein phosphorylation and destabilization, leading to the discovery of Prodigiosin (Wang et al., 2016) and KYA1797K (Cha et al., 2016). A recent paper has reported a novel β-catenin inhibitor MSAB, which directly binds to β-catenin, enhances its phosphorylation and increases its ubiquitination and proteasomal degradation (Hwang et al., 2016). Despite the significant progress in inhibiting β-catenin for cancer therapy, the development of β-catenin-specific targeting agents remains unsatisfactory. Because β-catenin is a multifunctional protein and even exerts opposite activity in different subcellular localizations (Kim et al., 2013), a complete inhibition of β-catenin has become an undesirable strategy for cancer therapy. Herein, novel approaches for specific inhibition of oncogenic β-catenin are still urgently needed.

Our data indicated that the β-carboline derivative SP141 specifically bound to β-catenin and induced its degradation without affecting the phosphorylated β-catenin expression. These results suggested that SP141 could function as an allosteric β-catenin inhibitor that induces its conformational changes and modulates the bindings of β-catenin with its regulators other than the destruction complex (Liu et al., 2001; Matsuzawa and Reed, 2001). It has been also reported that small molecule binding to the N-terminus of Armadillo repeat domain on β-catenin could induce β-catenin aggregation and degradation (de la Roche et al., 2012). Because SP141 was found to bind to the Armadillo repeat domain, it is also worth asking whether the formation of SP141-β-catenin complex causes the aggregation and degradation of β-catenin. As discussed above, the precise mechanisms for SP141’s inhibitory effects on β-catenin remain unclear, and an in-depth characterization of the SP141-β-catenin binding molecular mechanism is required in the future studies. Of note, the side effects, especially myelo- and gastrointestinal suppression in inhibiting Wnt/β-catenin pathway for cancer therapy have been reported (Blagodatski et al., 2014). The efficacy and safety of SP141 need to be evaluated in more clinically relevant models in our future studies.

In summary, this study demonstrates β-catenin as a molecular target of the carboline compound SP141. These results provide new insights into the strategic development of β-carboline derivatives as novel inhibitors of β-catenin. It is envisioned that SP141 can be a useful tool to explore the β-catenin and MDM2 dual-targeting approach for cancer therapy.

## Author Contributions

WW and RZ organized, conceived, designed, and supervised the study. J-JQ and XL designed and conducted the experiments, and drafted the manuscript. HD and JKB synthesized test compounds and helped study design and interpretation of data. All authors read and approved the manuscript.

## Conflict of Interest Statement

JKB, RZ, and WW hold a US patent for the use of β-carboline derivative in treating cancer. The other authors declare that the research was conducted in the absence of any commercial or financial relationships that could be construed as a potential conflict of interest.
