# Supplementary material for: Inhibiting β-Catenin by β-Carboline-Type MDM2 Inhibitor for Pancreatic Cancer Therapy
Source: Front Pharmacol. 2018 Jan 17;9:5. doi: 10.3389/fphar.2018.00005 (PMC5776119; doi:10.3389/fphar.2018.00005)
Supplement: Supplementary file 1 [file Data_Sheet_1.DOCX]

**Supplementary Information**

**Inhibiting β-Catenin by β-Carboline-type MDM2 Inhibitor for Pancreatic Cancer Therapy**

Jiang-Jiang Qin^1≠^, Wei Wang^1,2 ≠*^, Xin Li^1^, Hemantkumar Deokar^3^, John K. Buolamwini^3^

and Ruiwen Zhang^1,2*^

**Authors’ Affiliations:**

*^1^Department of Pharmacological and Pharmaceutical Sciences, College of Pharmacy, University of Houston, Houston, Texas 77204, USA*

*^2^ Center for Drug Discovery, University of Houston, Houston, Texas 77204, USA*

*^3^ Department of Pharmaceutical Sciences, College of Pharmacy, Rosalind Franklin University of Medicine and Science, 3333 Green Bay Road, North Chicago, IL 60064, USA*

**Supplementary Figures**

**Supplementary Figure 1. Original images of immunoblots in Figure 1A.**

**
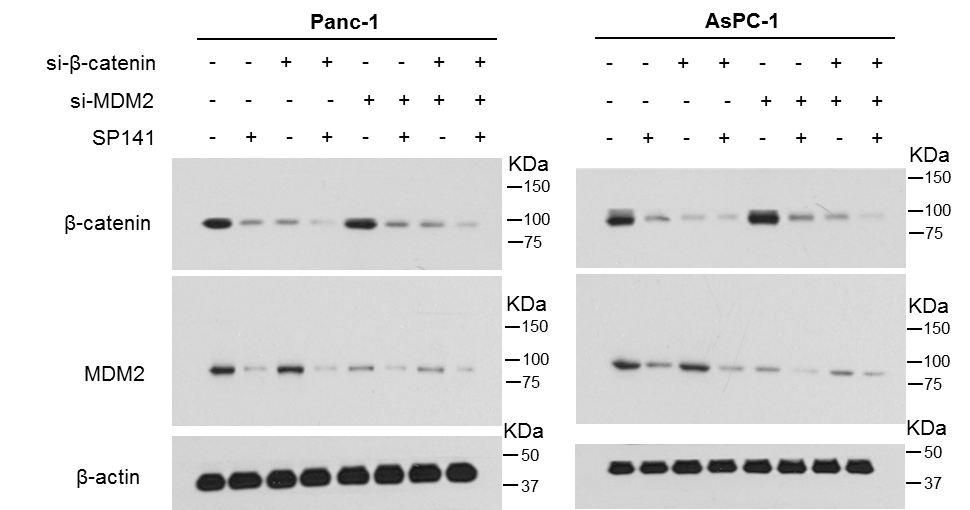
**

**Supplementary Figure 2. Original images of immunoblots in Figure 2A.**

**
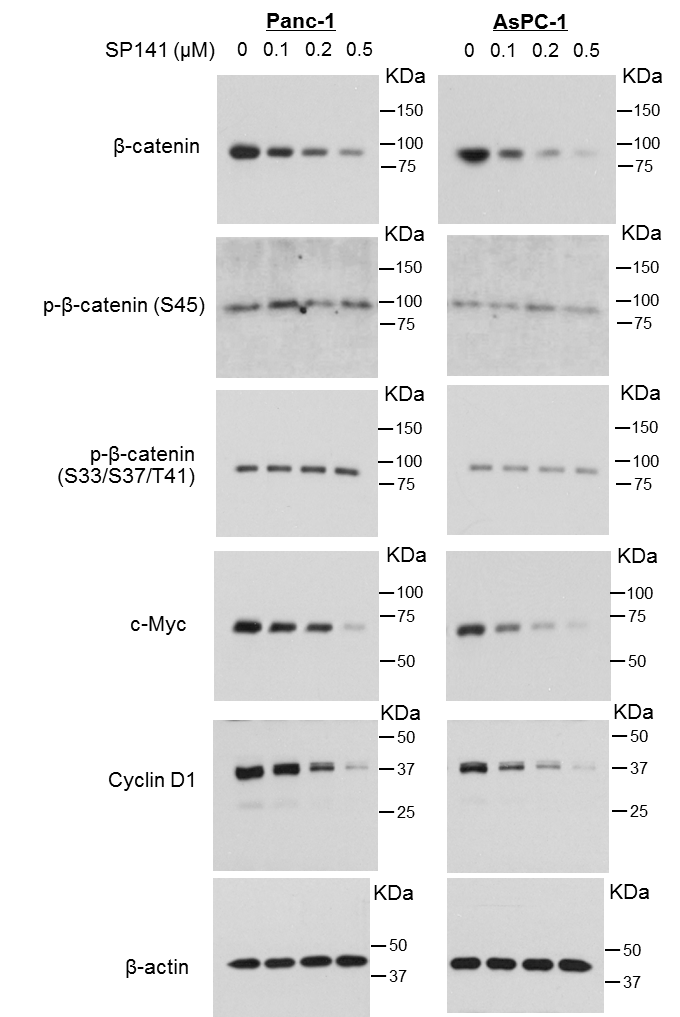
**

**Supplementary Figure 3. Original images of immunoblots in Figure 2C.**

**
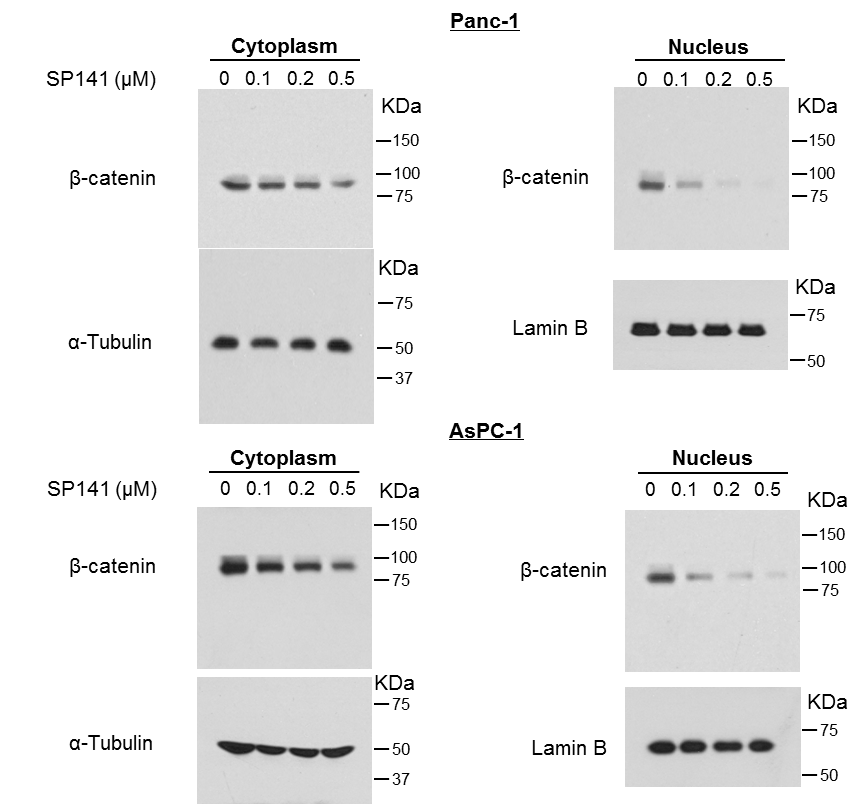
**

**Supplementary Figure 4. Original images of immunoblots in Figure 3B.**

**
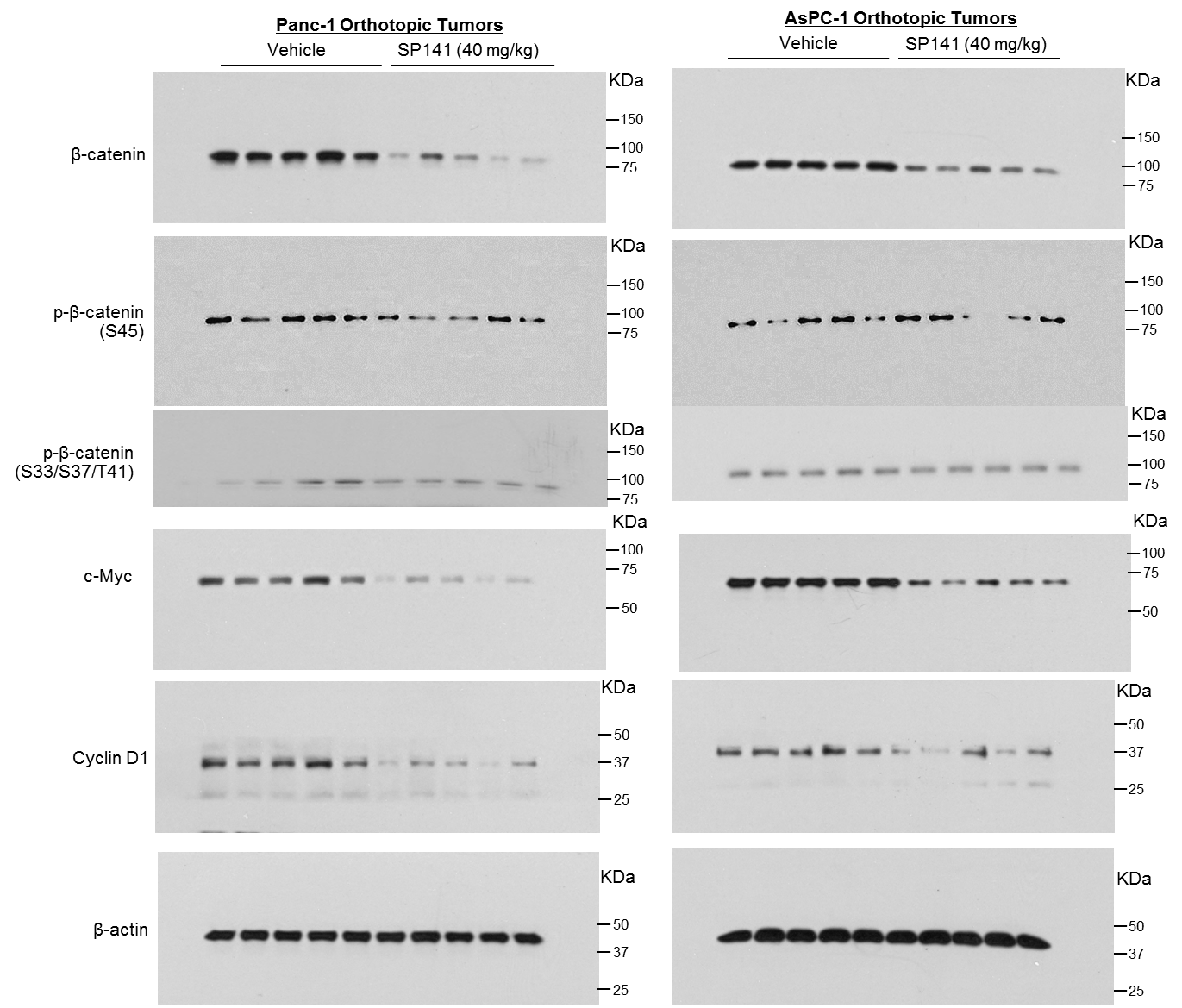
**

**Supplementary Figure 5. Original images of immunoblots in Figure 4A.**

**
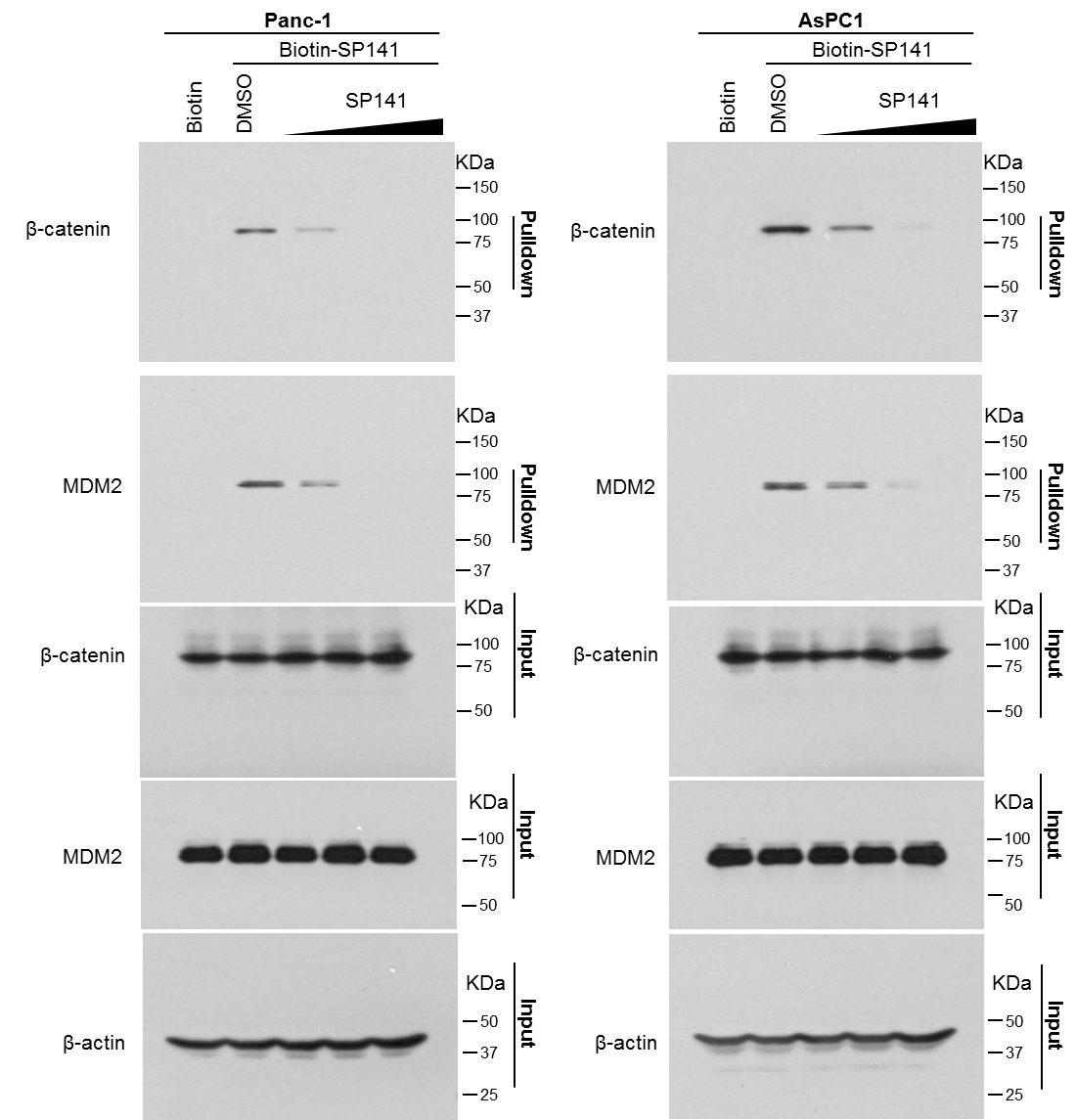
**

**Supplementary Figure 6. Original images of immunoblots in Figure 4B.**

**
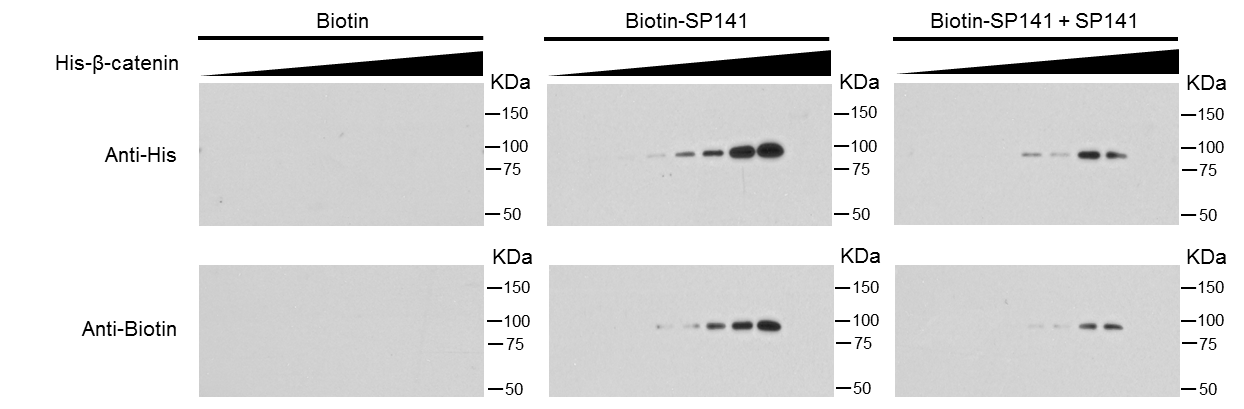
**

**Supplementary Figure 7. Original images of immunoblots in Figure 4E.**

**
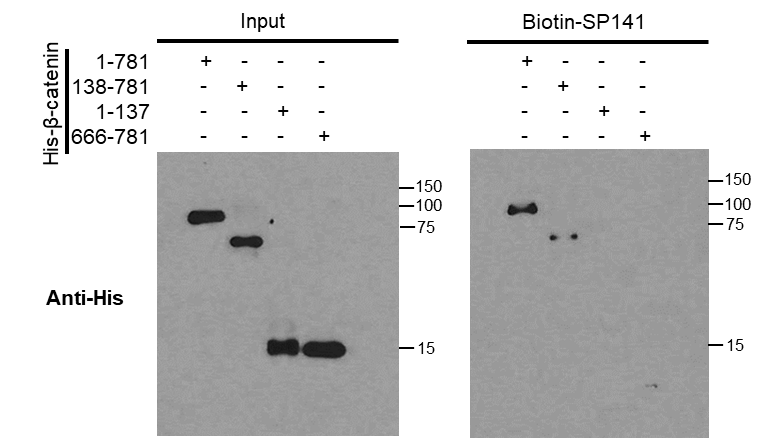
**

**Supplementary Figure 8. Original images of immunoblots in Figure 5A.**

**
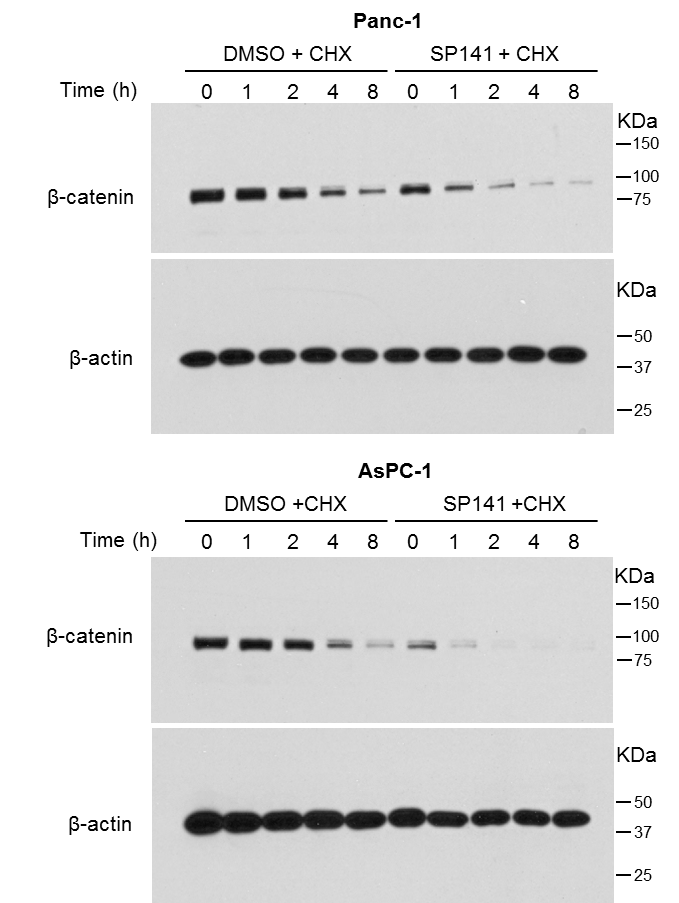
**

**Supplementary Figure 9. Original images of immunoblots in Figure 5B.**

**
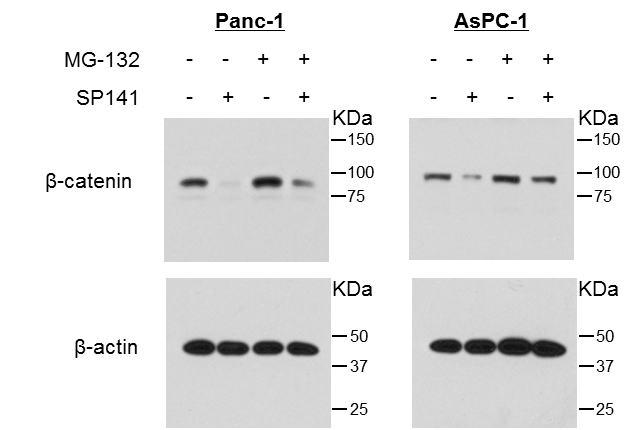
**

**Supplementary Figure 10. Original images of immunoblots in Figure 5C.**

**
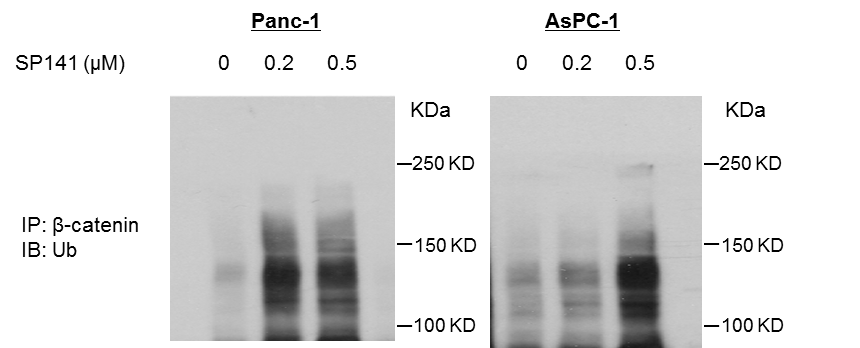
**

**Supplementary Figure 11. Original images of immunoblots in Figure 5D.**

**
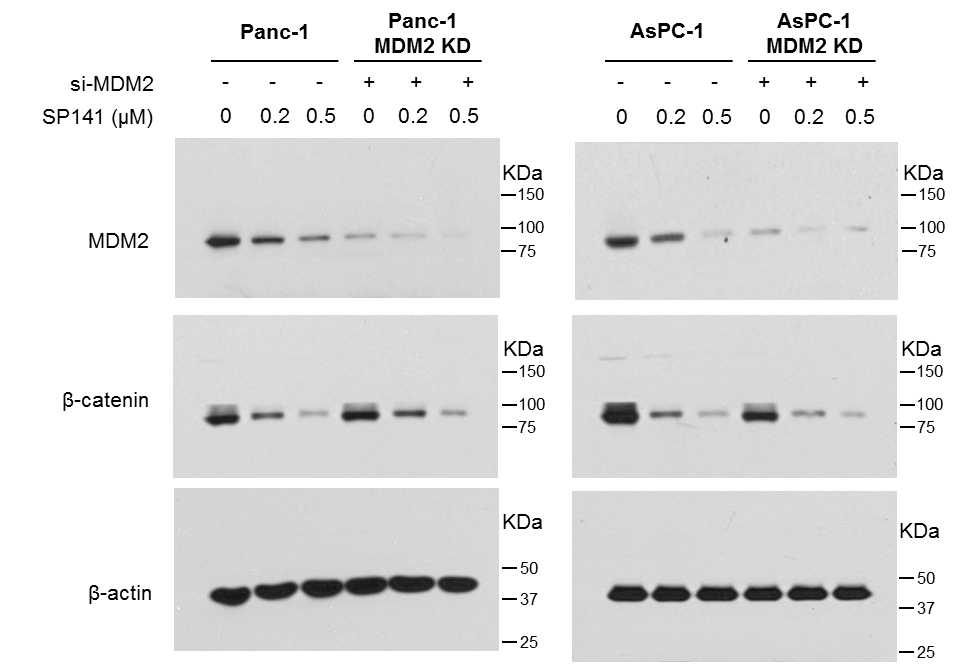
**
